# *In vitro* qualitative and quantitative CT assessment of iodinated aerosol nasal deposition using a 3D-printed nasal replica

**DOI:** 10.1186/s41747-019-0113-6

**Published:** 2019-08-21

**Authors:** Thomas Sartoretti, Manoj Mannil, Stefan Biendl, Johannes M. Froehlich, Hatem Alkadhi, Matthias Zadory

**Affiliations:** 1 KlusLab Research, Witikonerstrasse 15, CH 8032 Zürich, Switzerland; 2 Institute for Diagnostic and Interventional Radiology, University Hospital Zürich, University of Zürich, 8006 Zurich, Switzerland

**Keywords:** Aerosols, Contrast media, Iodine, Nasal sprays, Tomography (x-ray computed)

## Abstract

Computed tomography can provide high-resolution details on nasal anatomy being potentially useful for the assessment of nasal spray deposition. The purpose of this technical note was to present a method based on CT imaging to assess qualitatively and quantitatively the *in vitro* spray deposition patterns within the sinonasal cavities of a nasal replica obtained by three-dimensional (3D) printing, using iodinated contrast agent labelled solutions with high spatial and temporal resolution. Using a third generation dual-source CT scanner in single energy mode, scans of a nasal replica were acquired following application of iodinated contrast agent labelled aerosols with an iodine concentration of 92.5 mgl/mL. Two software programmes were then utilised (Osirix MD v.9.0, Pixmeo, Geneva, Switzerland; 3mensio, Pie Medical Imaging, Bilthoven, Netherlands) to generate three-dimensional reconstructions of the scans, thus enabling the rapid detection and visualisation of administered single droplets and their voxel-by-voxel localisation. Using this approach, we achieved recovery rates between 84–98% and 89–109% (depending on the software programme) of the total applied aerosol volume.

## Key points

- Computed tomography provides high-resolution details on nasal anatomy.
- Nasal aerosol deposition patterns can be imaged with computed tomography using iodinated contrast media
- Nasal aerosol deposition patterns can be quantified using a freeware software.

## Background

Transmucosal nasal drug delivery by means of nasal sprays is an option for challenging clinical situations offering systemic treatment approaches. Specifically, nasal drug delivery provides numerous advantages such as easy access, rapid onset of the therapeutic effect, no first-pass metabolism and potential for direct nose-to-central nervous system delivery [1–3].

Therefore, precise targeting of certain locations within the nasal cavity for efficient drug delivery is of interest explaining the decisive importance to better understand and characterise nasal spray deposition patterns. According to the current understanding, it is variable and influenced by several factors, specifically the high variability of the nasal anatomy between individuals, then by the patient’s administration technique itself and last but not least the technical characteristics of the nasal-spray system such as the plume angles, spray pressure or the colloid size [1–3]. This warrants the need for a systematic strategy to assess the nasal deposition characteristics of the pharmaceutically used devices both qualitatively and quantitatively with high precision. Moreover, the characterisation technique once validated *in vitro* should open up the pathways towards clinical endorsement.

Several nasal spray deposition assessment methods, both *in vivo* and *in vitro*, have been reported [1–5], yet each method bears substantial limitations such as lack of simplicity or low clinical suitability. An imaging approach such as computed tomography (CT) with its high temporal and spatial resolution and possibly low radiation dose may represent a valuable approach as it enables a fast acquisition of images and is readily available in various clinical settings [6–11].

The purpose of this study was to develop an *in vitro* approach based on CT imaging in combination with an iodinated contrast agent (ICA)-labelled nasal spray aqueous phase to qualitatively and quantitatively assess its deposition within the sinonasal cavity of a nasal replica.

## Methods

### ICA-labelled nasal spray

Our approach consisted of applying an ICA aerosol through a nose spray device into a nasal cavity replica followed by CT imaging. For the application of the iodinated solution, a specific type of unit dose spray device (Unit Dose, Aptar Pharma, Le Neubourg, France) was filled and utilised based on the supplier’s specifications, thus delivering with each puff a volume of 0.065 ± 0.010 mL of the iodinated solution into the nostrils. The sprays were labelled with a diluted aqueous ICA (Iopamidol DCI, Iopamiro, Bracco, Cadempino, Switzerland) solution with a concentration of 92.5 mgI/mL.

The ideal iodine concentration for optimal image impression was determined by preliminary CT scans of a dilution series of the IVA ranging from 0 to 370 mgI/mL iodine concentration using a nasal cavity replica. The optimal concentration was chosen so that no artefacts were visible on the CT images, while retaining suitable contrast for quantification.

### Nasal cavity replica

The nasal cavity replica made of synthetic polymer (epoxy resin) was prepared by means of stereolithography based on three-dimensional files of a 33-year-old female cadaver without nasal disease [12]. The resolution was quoted to be at about 0.15 mm. The material was chosen as it allows good distinction between the hyperdensity of ICA deposited droplets and the x-ray imaging of the anatomical structure of the cast.

**Fig. 1:**
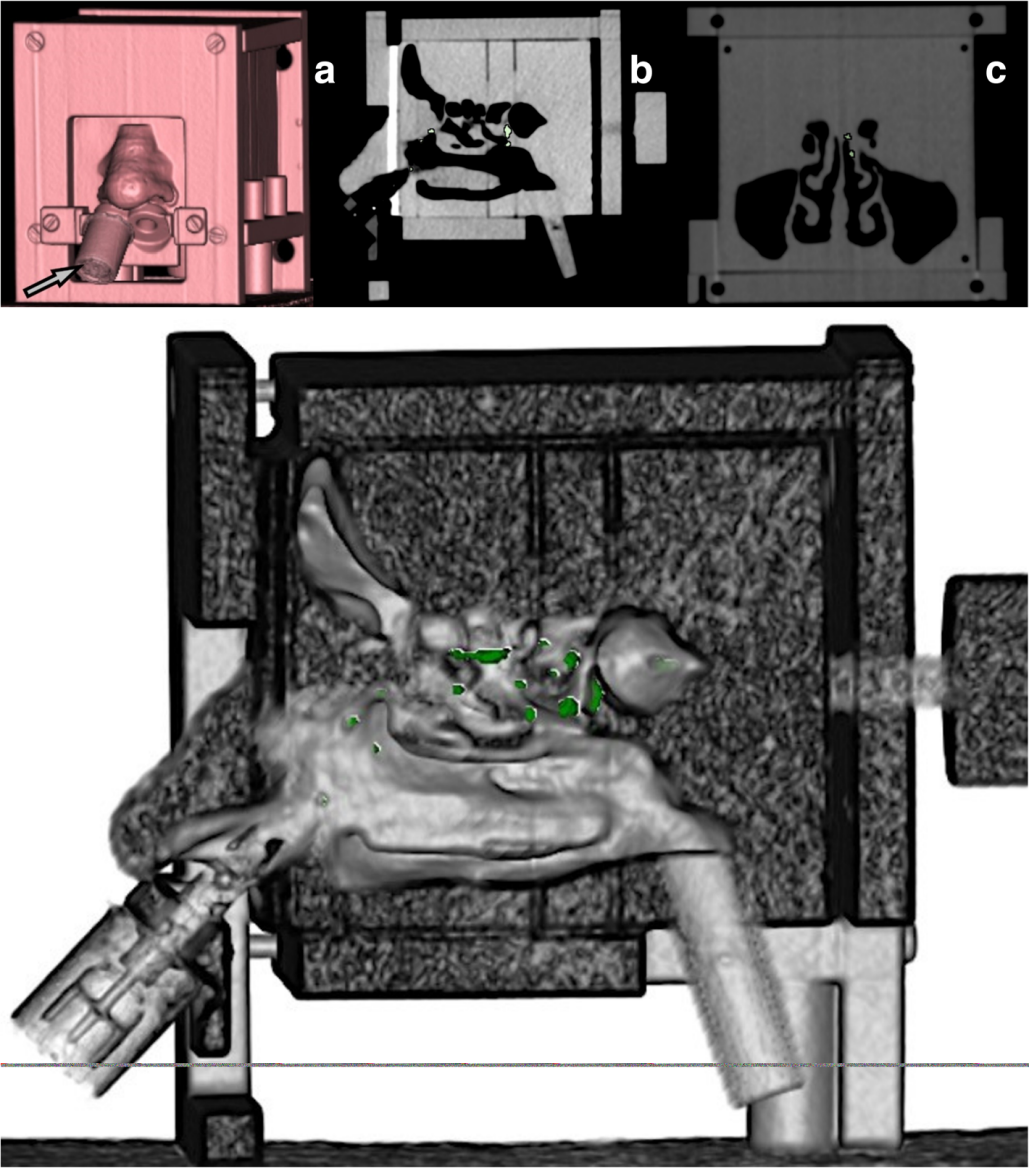
Three-dimensional volume-rendered computed tomography image demonstrating the nasal cast from anteriorly: the nose spray (arrow) was applied into the support below the right nostril for standardised application of the spray (**a**). Sagittal reformation depicting the iodinated contrast agent droplets in the nasal cavity (bright hyperdense spots) (**b**). The colours indicate pixels with attenuation values exceeding the predefined threshold in the region of interest (**c**). On the lower image, a sagittal image after segmentation is shown. The green colour indicates the iodinated deposited droplets. Note the depositions mainly in regions next to the superior and middle concha

The nose of the replica with a special support was constructed of a flexible polymer (silicone) to allow for proper placement of the nasal application device. Specifically, the spray device was always placed into a dedicated support below the nostril to allow for a standardised application and positioning (45° angle) of the spray system (Fig. 1a).

### CT scan protocol and aerosol application

For all experiments, a third generation dual-source CT scanner (SOMATOM Force, Siemens Healthineers, Forchheim, Germany) in single energy mode was utilised. Scan parameters are shown in Table 1. Initially, an unenhanced scan of the nasal replica without application of the aerosol was performed (native baseline, serving as internal control) followed by four applications of the aerosol: two single applications into the left nostril, one single application into the right nostril, one further application on the right side consisting of two single applications in rapid succession, thus totalling a double-dose. For each application, the spray device was positioned into one nostril, secured by the dedicated support, and a puff was applied by pressing manually the triggering mechanism on the bottom of the device. The replica was always scanned in the exact same position simulating the position of the head of an upright subject. CT scans were acquired on average 10 ± 3 s after device-controlled application of the iodinated solution into the nostrils of the replica. After scanning, the replica was disassembled, washed, and dried thoroughly followed by a non-enhanced scan before execution of the next exposition series (Table 2).

**Table 1:** Scan protocol and reconstruction parameters using a dual-source computed tomography scanner

Parameters	Values
Tube voltage (kVp)	90
Tube current (mAs)	67–98*
Spiral pitch factor	0.7
Scan time (s)	6.8
CTDI_vol_ (mGy)	7.6
Slice thickness (mm)	0.75
Increment (mm)	0.5
Field of view (mm)	350 × 1200
Matrix size	512 × 512
Pixel size (mm)	0.488 × 0.488
Scan orientation	Coronal

*CTDI*_*vol*_ volume computed tomography dose index. The unit was a Somatom Force, Siemens Healthineers, Forchheim, Germany

*Attenuation-based tube current modulation

**Table 2:** Overview of the volumetric quantification and recovery rates of the nasal droplet depositions after nasal application of an iodinated contrast agent solution with 92.5 mgI/mL using a single-dose nasal spray device

Application	Applied volume of iodinated aerosol (cm^3^)	Volume quantified using OsirixMD (cm^3^)	Volume quantified using 3mensio (cm^3^)	Recovery rate (%) (OsirixMD; 3mensio)
1	0.065	0.0595	0.058	91.5; 89.2
2	0.065	0.0638	0.071	98.1; 109.2
3	0.065	0.0545	0.061	83.8; 93.8
4	0.130	0.1090	0.118	83.8; 90.7

### CT data post-processing

Post-processing was performed on a personal computer without dedicated graphics processing unit by two readers (J.F. 20 years of experience; M.Z., 4 years of experience.). First, a three-dimensional (3D) reconstruction of CT images was performed using a freeware postprocessing software (Osirix MD v.9.0, Pixmeo, Geneva, Switzerland). An optimal subjective image quality was achieved by manually optimising the contrast between hyperdense droplets and less intense structures of the replica enabling qualitative assessments (Fig. 1b, c). Second, using two different software programmes (Osirix MD v.9.0, Pixmeo, Geneva, Switzerland; 3mensio, Pie Medical Imaging, Bilthoven, Netherlands), quantification of the deposition within the sinonasal cavity of the nasal replica was performed (Fig. 1). Both programmes allowed for the direct identification of droplet depositions on the scans performed after the application of the iodinated solution by using a threshold approach based on the density values of the voxels, expressed in Hounsfield units (HU). The total deposition volume was then calculated fully automatically with a region-growing algorithm. To determine the accuracy, the recovery rate was calculated by dividing the measured volume (region-growing algorithm) through the applied volume multiplied by 100.

### Radiation dose estimation

Radiation dose estimations were taken from the electronically logged dose protocols which are automatically generated by the scanner after data acquisition. The average volume CT dose index (CTDI_vol_) per scan was 7.6 mGy.

## Results

### Qualitative analysis

The iodinated droplets were visible as single speckles (rather than large clouds) adhering to the underlying anatomical structures (Fig. 1). A good contrast between droplets and cast was achieved because of the large differences in attenuation between droplets (mean of 3 measurements, 1080 HU) and cast (mean values of 480 HU).

**Fig. 2:**
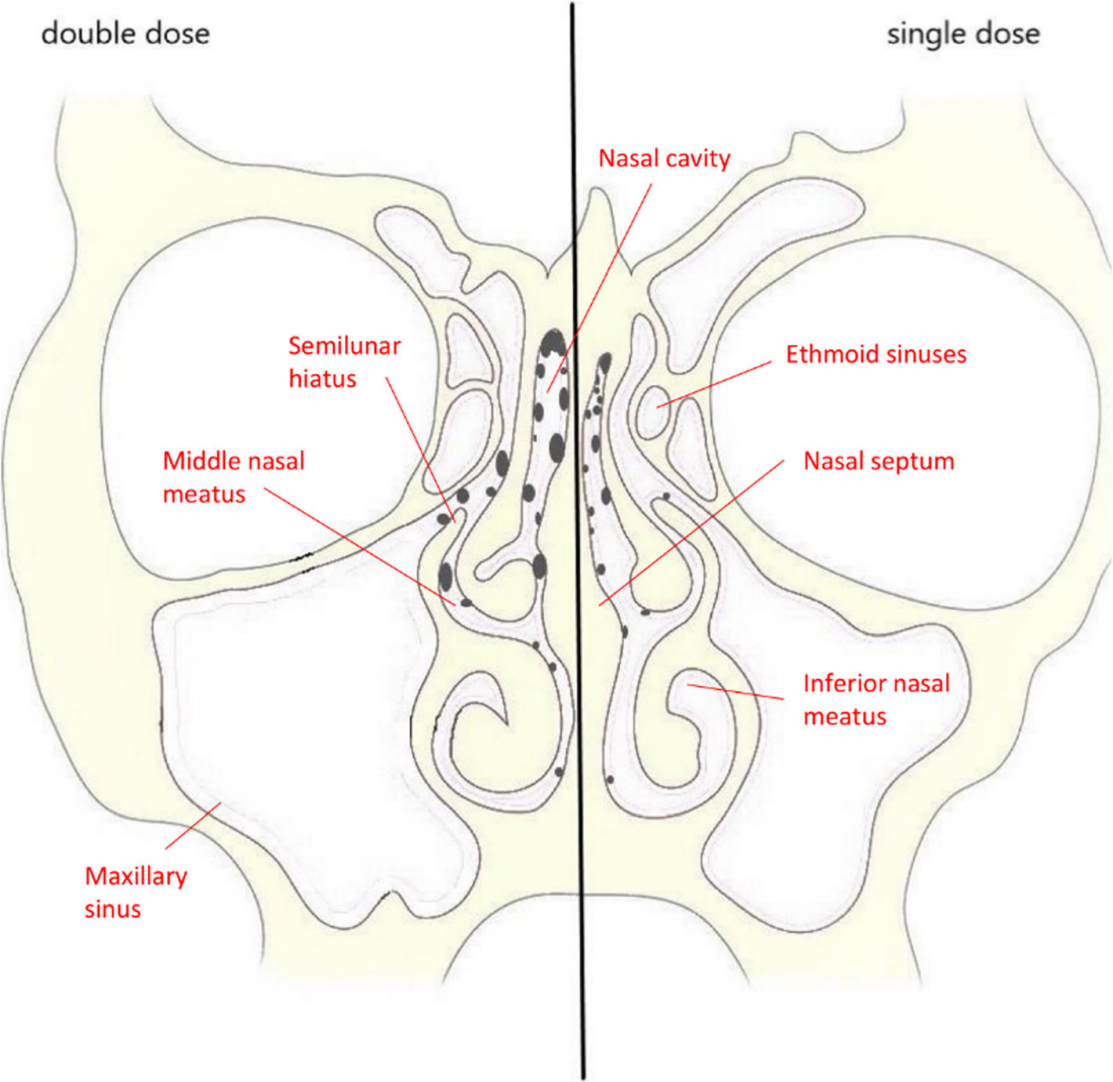
Coronal projection diagram of the distribution of droplets within the nasal cavity. The left side shows the distribution pattern after nasal application of the double dose (two single applications in rapid succession) while the right sides represent a single dose application. The distribution of droplets from different coronal slices (including both single and double dose scans) was projected onto a single coronal slice. Droplets are indicated as dark spots. The droplets’ size is an accurate representation of their relative size in comparison to the underlying anatomical structures. Note the presence of droplets predominantly in the superior and middle nasal meatus and for the double dose in the regions of the semilunar hiatus

Regarding the spatial distribution in the nasal cavity of the single nasal spray device applications, we found large parts of the droplets in the upper and middle nasal meatus (Fig. 2). Only very few droplets were visible in the inferior part of the nasal cavity despite gravitational influence, and no droplets were visible in distant regions close to the trachea. No side-dependent difference in the distribution of the droplets could be observed when comparing left *versus* right side application. The deposition patterns between the left and right sides (and between different scans) were similar.

After the application of the double dose (administered as two subsequent single doses) into the right nostril, larger single droplets were visible in the upper nasal meatus, a larger number of droplets were visible in the region near the semilunar hiatus and very few droplets reached more distal regions of the nasal cavity.

### Quantitative analysis

Quantification yielded recovery rates ranging from 83.8 to 98.1% of the total applied volume (0.065 mL or 0.13 mL) with OsirixMD and from 89.2 to 109.2% of the total applied volume (0.065 of 0.13 mL) with 3mensio, respectively. OsirixMD achieved the highest accuracy in quantifying the deposition pattern of the second single application into the left nostril (0.0638 of 0.065 mL, 98.1%) while 3mensio achieved the highest accuracy in quantifying the deposition pattern of the single application into the right nostril (0.061 of 0.065 mL, 93.8%). Detailed results are provided in Table 2.

## Discussion

Our study is the first, to our knowledge, to demonstrate the feasibility of CT imaging to enable a qualitative and quantitative assessment of *in vitro* nasal spray droplet deposition after nasal administration. This not only promises to improve characterisation of the various available nasal spray devices and their deposition patterns, but also opens up the field for this promising new drug administration route in the systemic treatment of challenging pathologies. Based on preliminary CT scans, we found that the qualitative assessment was adequate at iodine concentrations of 80 mg/mL and higher. For the quantitative assessment, it seems that the highest possible concentration without artefacts should be chosen. At 92.5 mgI/mL iodine concentration, a good trade-off was achieved, yielding total recovery rates between 84–98% and 89–109%, respectively, of the total applied aerosol volume depending on the post-processing software used.

Currently, scintigraphy with a radioactive aerosol is considered the reference standard for nasal deposition studies [5]. Several drawbacks such as the rather slow two-dimensional imaging with a low spatial resolution, lack of anatomical visualisation, and the use of radioactive tracers [3, 4] can be overcome with a CT-based method. Imaging units combining single-photon emission computed tomography and CT provide improved resolution and high sensitivity, offering the opportunity to visualise the underlying anatomical structures [4], but still suffers from long imaging times and the need of administering a radioactive tracer such as ^99m^Tc (400 MBq of DTPA-Technetium 99 m (^99m^Tc) solution suffices to map the aerosol deposition using the combined single-photon emission computed tomography and CT approach described by Leclerc et al. [4]).

We showed here that a pure CT-based imaging technique using an ICA labelling allows for rapid detection of administered single droplets and their voxel-by-voxel localisation, potentially overcoming those drawbacks of the current reference standard from nuclide imaging. Instead of deposition patterns provided by using radioactive tracers resembling intranasal clouds, our method enables visualisation of single droplets before the depositions aggregate into larger speckles under gravitational influence. This might be explained by keeping the duration between aerosol application and image acquisition short (10 s). Moreover, our method using a low dose clinical CT protocol (CTDI_vol_ 7.6 mGy) can most probably be easily transferred to a clinical setting.

We envisage the future use of this method in a personalised medicine setting, where nasal drug delivery is tailored to the needs of individual patients and more precise dose regimens are aimed for. Specifically, this approach could be used to determine the optimal application angle of nasal sprays for individual patients taking into account certain anatomical configurations of the nasal cavity, which would enable optimal drug delivery to specified locations. The systemic availability of the applied drugs and precise dosing highly depend on the deposition patterns emphasising the importance of this method. Moreover, this could be of interest also for patients with neurological disorders relying on medication that can pass directly from the upper nasal cavity to the central nervous system via the olfactory regions.

There are several limitations to our study that have to be acknowledged. In the CT scan protocol (designed for clinical use), a slice thickness of 0.75 mm with 0.5 mm increment was chosen. We assume that resolution and other protocol parameters may significantly influence the quantification process and its accuracy. Despite achieving high recovery rates, it needs to be emphasised that it is too early to estimate the overall accuracy and reliability of our quantification methods. The differences in recovery rates observed between the two software programmes may stem from different algorithms and thresholding approaches. Specifically, partial volume effects may explain why we observed recovery rates greater than 100%. Voxels containing even miniature amounts of iodine still exhibit high average HU values due to the large difference in density between iodine and air (or the cast material). Thus, even slight thresholding differences between the two software programmes may lead to the inclusion or exclusion of vast amounts of voxels containing very little amounts of iodine. In case of recovery rates greater than 100%, the algorithm probably overestimated the amount of iodine by selecting a slightly lower threshold than the other software programme.

We chose two established and simple-to-use freeware software programmes for quantification, yet other software solutions may yield different results. These factors besides the use of other nasal replicas should be examined systematically in future studies. Furthermore, when utilising this method to accurately characterise deposition patterns of individuals in future studies, inspiration through the nose should probably be included into the simulation eventually using a vacuum [13] as this may significantly impact the deposition pattern. Further quantification methods allowing better differentiation of materials including dual-energy CT techniques should be investigated.

In conclusion, the present *in vitro* study indicates that CT-based imaging allows for a qualitative and quantitative assessment of the distribution of ICA-labelled nasal spray solutions administered into the nasal cavity with both high spatial and temporal resolution.

## Abbreviations

3D: Three-dimensional
CT: Computed tomography
CTDI_vol_: Volume CT dose index
HU: Hounsfield units
ICA: Iodinated contrast agent
